# Dual Inhibition of Myc Transcription and PI3K Activity Effectively Targets Colorectal Cancer Stem Cells

**DOI:** 10.3390/cancers14030673

**Published:** 2022-01-28

**Authors:** Miriam Gaggianesi, Laura Rosa Mangiapane, Chiara Modica, Vincenzo Davide Pantina, Gaetana Porcelli, Simone Di Franco, Melania Lo Iacono, Caterina D’Accardo, Francesco Verona, Irene Pillitteri, Alice Turdo, Veronica Veschi, Ornella Roberta Brancato, Giampaolo Muratore, Giuseppe Pistone, Maria Rita Bongiorno, Matilde Todaro, Ruggero De Maria, Giorgio Stassi

**Affiliations:** 1 Department of Surgical, Oncological and Stomatological Sciences (DICHIRONS), University of Palermo, 90127 Palermo, Italy; miriam.gaggianesi@unipa.it (M.G.); chiara.modica@unipa.it (C.M.); vincenzodavide.pantina@unipa.it (V.D.P.); simone.difranco@unipa.it (S.D.F.); melania.loiacono@unipa.it (M.L.I.); irene.pillitteri@policlinico.pa.it (I.P.); veronica.veschi@unipa.it (V.V.); ornellaroberta.brancato@community.unipa.it (O.R.B.); giampaolo.muratore@unipa.it (G.M.); 2 Department of Health Promotion Sciences, Internal Medicine and Medical Specialties (PROMISE), University of Palermo, 90127 Palermo, Italy; laurarosa.mangiapane@unipa.it (L.R.M.); gaetana.porcelli@unipa.it (G.P.); caterina.daccardo@unipa.it (C.D.); francesco.verona@unipa.it (F.V.); alice.turdo@unipa.it (A.T.); giuseppe.pistone@unipa.it (G.P.); mariarita.bongiorno@unipa.it (M.R.B.); matilde.todaro@unipa.it (M.T.); 3 Dipartimento di Medicina e Chirurgia Traslazionale, Facoltà di Medicina e Chirurgia, Università Cattolica del Sacro Cuore, 00168 Roma, Italy; 4 Fondazione Policlinico A Gemelli IRCCS, 00168 Roma, Italy

**Keywords:** anti-tumor drug resistance, colorectal cancer, combination therapies, cancer stem cells

## Abstract

**Simple Summary:**

Compelling evidence has shown that cancer stem cells (CSCs) are responsible for high resistance to conventional anti-cancer therapies. Here, we demonstrate that the tumor microenvironment protects CR-CSCs from EGFR/HER2, BRAF and PI3K targeting, promoting CD44v6 and Myc expression. Alternatively, as a substitution for HER2 and BRAF, the Myc transcription inhibitor can overcome the protective effects of microenvironmental cytokines, impairing the survival of CR-CSCs. These data highlight the targeting of Myc and PI3K activity as a novel therapeutic strategy against advanced colorectal cancer.

**Abstract:**

Despite advances in the curative approach, the survival rate of advanced colorectal cancer (CRC) patients is still poor, which is likely due to the emergence of cancer cell clones resistant to the available therapeutic options. We have already shown that CD44v6-positive CRC stem cells (CR-CSCs) are refractory toward standard anti-tumor therapeutic agents due to the activation of the PI3K pathway together with high HER2 expression levels. Tumor microenvironmental cytokines confer resistance to CR-CSCs against HER2/PI3K targeting by enhancing activation of the MAPK pathway. Here, we show that the CSC compartment, spared by BRAF inhibitor-based targeted therapy, is associated with increased expression levels of CD44v6 and Myc and retains boosted clonogenic activity along with residual tumorigenic potential. Inhibition of Myc transcription, downstream of the MAPK cascade components, and PI3K pathway activity was able to overcome the protective effects of microenvironmental cytokines, affecting the survival and the clonogenic activity of CR-CSCs, regardless of their mutational background. Likewise, the double targeting induced stabilization of mouse tumor avatars. Altogether, these data outline the rationale for dual kinase targeting of CR-CSCs to prevent their adaptive response, which would lead to disease progression.

## 1. Introduction

Colorectal cancer (CRC) is the second leading cause of cancer-associated death worldwide [1]. Thanks to the advances in early diagnosis and treatments, the 5-year survival rate for CRC patients with localized disease now reaches about 90%. Nevertheless, the percentage drops to 14% for patients diagnosed with metastatic lesions [2]. Mutations in the Wnt/β-catenin, PI3K/AKT and MAPK pathways lead to their aberrant activation, which drives CRC initiation and progression [3,4]. Compelling evidence has demonstrated that a subset of cancer cells, named cancer stem cells (CSCs), are characterized by a tumor initiation ability and are responsible for recurrence and anticancer drug resistance [5,6]. These CSC peculiarities are sustained by (i) deregulation of key signaling pathways implicated in normal stem cell maintenance, such as WNT, Hedgehog and Notch; (ii) high expression of ABC transporter and anti-apoptotic factors; and (iii) proficient DNA repair machinery [7,8]. The WNT signaling pathway is essential to maintain the homeostasis of colonic crypts, regulating turnover and differentiation of intestinal stem cells (ISCs). Hence, this pathway is aberrantly activated in many tumors and about 90% of CRCs harbor mutations in WNT pathway components [9,10]. We demonstrated that metastatic CR-CSCs express increased levels of CD44v6 and display high Wnt activity [11]. Moreover, cytokines released by the tumor microenvironment (TME) boost CD44v6 expression and WNT signaling activation [11,12]. Although much effort has been directed toward developing targeted therapies that are able to interfere with CRC-related signaling pathways, only a few drugs have successfully advanced from preclinical tests to clinical trials. The reported results of several clinical trials (NCT00154102, NCT00297271, NCT01228734) highlighted that patients bearing *BRAF^V600E^* and *RAS* mutations do not benefit from treatment with anti-EGFR therapies [13,14,15,16]. Recently, we demonstrated that about 50% of *BRAF* and *RAS* wt CRC sphere cells (CR-CSphCs) are resistant to cetuximab treatment due to high expression levels of CD44v6 [11,17]. Many other therapeutic strategies, including anti-angiogenic drugs and Met pathway and immune checkpoint inhibitors, are currently under evaluation for managing CRC treatment [2,18,19,20,21]. Another common mechanism of resistance to standard and targeted therapies is gene amplification/overexpression [22,23,24]. The *MYC* oncogene is deregulated in 70% of cancers, including CRC, and amplified in about 20% of CRC patients [25,26]. Increased Myc expression levels are associated with the onset of tumor growth, constitutive activation of Wnt/β-catenin and an impaired survival rate in CRC patients. Numerous shreds of evidence have demonstrated that a chemoresistant phenotype characterizes tumors expressing high levels of Myc. Likewise, cancer cells surviving the treatment with standard chemotherapeutic compounds displayed higher *MYC* expression levels [27]. Resistance to Met inhibitors in *MET*-addicted gastric cancer cell lines is sustained by Myc, and the double blockade of Met and Myc can lessen the tumor growth of *MET*-amplified patient-derived xenografts tumors [28]. In *KRAS* mutant CRC cells, the high expression of Myc confers resistance to HDAC inhibitor SAHA [29]. All these data point out that, despite advances in the improvement of targeted therapies for advanced CRC, treatment options for RAS mutated patients remain a challenge.

We previously demonstrated that CD44v6^+^ CR-CSCs are highly resistant to standard therapies and can be efficiently targeted by PI3K inhibitors only in the absence of a protective TME. Cytokines released by cancer-associated fibroblasts (CAF) counteract the anti-tumoral effect of HER2/PI3K targeting via the upregulation of the MAPK pathway, as indicated by the ability of the MEK inhibitor-based combination to induce tumor shrinkage [17,30]. Here, we demonstrate that the combinatorial treatment of anti-EGFR/HER2, BRAF and PI3K inhibitors induces the selection of a CD44v6^+^/WNT^high^ cell population that displays high expression levels of Myc. The dual indirect targeting of CD44v6 and Myc, using PI3K and cyclin-dependent kinase (CDK) inhibitors, reduces, even in the presence of a TME, the survival and clonogenic activity of CR-CSphCs, regardless of the mutational background and/or *MYC* gene amplification, and stabilizes the growth of tumor xenografts.

## 2. Materials and Methods

### 2.1. Isolation and Maintenance of CR-CSphCs and CAFs

Colorectal cancer sphere cells (CR-CSphCs) were obtained from 43 human tumor tissue specimens following the Human Experimentation Policy of Policlinico “Paolo Giaccone”, Palermo (authorization CE9/2015), as previously described. CRC specimens were first minced into small pieces using surgical scissors and then enzymatically digested with collagenase (0.6 mg/mL Sigma-Aldrich, St. Louis, MO, USA) and hyaluronidase (10 µg/mL, Sigma-Aldrich, St. Louis, MO, USA) in DMEM medium with continuous shaking (150 rpm) at 37 °C for 30 min. Thereafter, the obtained suspension was centrifuged at 200× *g* for 5 min and the cell pellet resuspended in serum-free stem cell medium (SCM) supplemented with recombinant human FGF-basic (100 ng/mL, Peprotech, Cranbury, NJ, USA) and recombinant human EGF (50 ng/mL, Peprotech, Cranbury, NJ, USA) (see also [31]). Cells were seeded in ultra-low attachment culture flasks at a confluence of 1 × 105 cells/mL. Cell propagation was accomplished, dissociating sphere cells with Accutase (ThermoFisher Scientific, Waltham, MA, USA) when they reached a diameter of 50–100 µM. All the experiments were performed with early-passage CR-CSphCs (<20).

CRC spheres were authenticated by comparing them with their patient-related tumor tissues in an analysis of repeated polymorphisms (GlobalFiler™ STR kit, Applied Biosystem, Thermo Fisher Scientific, Waltham, MA, USA) using an ABIPRISM 3130 genetic analyzer (Applied Biosystem, Thermo Fisher Scientific, Waltham, MA, USA). Moreover, every 2 months, CR-CSphC cultures were tested for mycoplasma contamination (MycoAlert^TM^ Plus Mycoplasma Detection Kit, Lonza, Houston, TX, USA). The mutational profiles of the CR-CSphCs were analyzed with a targeted DNA custom panel by sequencing them with the MiSeq platform (Illumina, San Diego, CA, USA). *MYC* copy number variation (CNV) was evaluated using Digital Droplet PCR (Bio-Rad QX200 reader, Bio-Rad, Hercules, CA, USA) according to the manufacturer’s instructions. The obtained data were normalized with the *EIF2C1* gene and a *MYC/EIF2C1* ratio ≥4 was used to identify amplified *MYC* samples [32]. The presence of microsatellite instability (MSI) was assessed with a GENEQUALITY CC-MSI kit according to the manufacturer’s instruction (AB Analitica, Padova, Italy). The presence of four or more unstable markers identified high MSI samples and one to three identified low MSI samples; the absence of unstable markers identified stable samples (Table 1).

CAFs were isolated from CRC samples and plated in DMEM medium supplemented with 10% fetal bovine serum (FBS) in an adherent condition. CAFs were detached using Trypsin-EDTA solution when they reached 70–80% confluence and were cultured for at least 10 passages.

### 2.2. CR-CSphCs In Vitro Treatment

CR-CSphCs were exposed to vemurafenib (1 µM, S1267 Selleckchem, Houston, TX, USA), cetuximab (20 µg/mL) and trastuzumab (10 µg/mL) (provided by Policlinico “Paolo Giaccone”, Palermo, Italy), as well as BKM120 (1 µM, S2247 Selleckchem, Houston, TX, USA), dinaciclib (10 nM, S2768 Selleckchem, Houston, TX, USA) and taselisib (1 µM, GDC-0032 Selleckchem, Houston, TX, USA). All the compounds, with the exception of dinaciclib, were refilled every 48 h. To evaluate the significance of the dinaciclib and taselisib combinatorial treatment, CR-CSphCs were exposed to different concentrations of dinaciclib (5, 10 and 25 nM) and taselisib (0.2, 0.5 and 1 µM) for 72 h and the data were analyzed with Synergy Finder software (version 2.0) [33] (Table 2). To obtain CAF-conditioned medium (CAF CM), CAFs were grown to subconfluence and maintained in SCM for 48 h. Before addition to the CR-CSphCs, the CAF CM was filtered with a 0.22 µm filter to remove any cell debris.

### 2.3. Clonogenic, Sphere-Forming and Colony-Formation Assays

The clonogenicity of CR-CSphCs, previously treated as indicated, was assessed by plating them on ultra-low attachment 96-well plates at the single-cell level. SCM was refreshed once a week, and the results were statistically evaluated after 21 days. The sphere-forming assay was performed by culturing xenograft-derived sphere cells at clonal density (1 × 10^3^ cells/mL), avoiding cell–cell contact. Sphere-formation capacity was measured by counting the number of cell clusters that reached the diameter of ≥50 µm after ten days of culture. For the colony-forming assay, 2 × 10^3^ CR-CSphCs were seeded on low-melting agarose (Agarose Sea Plague Agar, Invitrogen, Thermo Fisher Scientific, Waltham, MA, USA) and maintained for 21 days. Crystal violet staining (0.01%) was used to reveal cell colonies and the clonogenic potential of CR sphere cells was assessed using ImageJ software (Bethesda, MD, USA).

### 2.4. Cell Viability

CR-CSphC viability was evaluated with the CellTiter 96^®^ Aqueous One Solution Cell Proliferation Assay (MTS, Promega, Madison, WI, USA) and, after 2 h, the variation in absorbance was measured with a GDV MPT 370 reader (DV 990 BV6). To validate the obtained data, the percentage of live cells was calculated with the trypan blue exclusion test.

### 2.5. Lentiviral Production and Transduction

FOP-GFP.mC (Addgene, Watertown, MA, USA, 35492) or TOP-GFP.mC (Addgene, Watertown, MA, USA, 35491) lentiviral supernatants were obtained following the protocol described in [31] and concentrated with the Lenti-X Concentrator reagent (Clontech, Takara Bio, San Jose, CA, USA). A total of 1 × 10^5^ CR-CSphCs were exposed to the obtained lentiviral supernatants with the addition of 8 μg/mL of polybrene (Sigma-Aldrich, St. Louis, MO, USA).

### 2.6. Flow Cytometry and Cell Sorting

CR-CSphCs were resuspended in PBS and incubated with conjugated antibodies CD44v6-APC (2F10, R&D system, Minneapolis, MN, USA) or the corresponding isotype-matched control (IC002A, mouse IgG1, R&D system, Minneapolis, MN, USA) for 1 h at 4 °C, and 7-AAD staining was used to exclude dead cells (0.25 μg/1 × 10^6^ cells, BD Biosciences, Franklin Lakes, NJ, USA). To enrich TOP-GFP and CD44v6 cell fractions, CR-CSphCs were resuspended in 2% BSA 2 mM EDTA PBS and filtered using a 70 µm mesh, with cell sorting performed with a FACS Melody cell sorter (BD biosciences, Franklin Lakes, NJ, USA).

### 2.7. Western Blot

Total proteins from CR-CSphCs were isolated as previously described [17]. After performing SDS-PAGE, proteins were blotted on nitrocellulose membranes. Membranes were incubated with blocking solution (5% Blotto, nonfat dry milk, Santa Cruz Biotechnology, Dallas, TX, USA, PBS 0.1% Tween 20) for 45 min at R.T. and then exposed at 4 °C for 16 h with antibodies against phospho-AKT xp (Ser473; D9E, rabbit, IgG, CST, Danvers, MA, USA), AKT (rabbit polyclonal, CST, Danvers, MA, USA), phospho-MEK1/2 (Ser217/221; 41G9, rabbit IgG, CST, Danvers, MA, USA), MEK1/2 (rabbit polyclonal, CST, Danvers, MA, USA), phospho-ERK 1/2 (Thr202/Tyr204; rabbit polyclonal, CST, Danvers, MA, USA), ERK 1/2 (137F5, rabbit IgG, CST, Danvers, MA, USA) or Myc (rabbit polyclonal, CST, Danvers, MA, USA). After incubation with anti-mouse or anti-rabbit HRP-conjugated secondary antibody (goat IgG; Thermo Fisher Scientific, Waltham, MA, USA), chemiluminescence signals were detected with Amersham imager 600 (GE Healthcare, USA). The densitometric analysis of β-actin (8H10D10, mouse, CST, Danvers, MA, USA)-normalized protein levels was undertaken with ImageJ software.

### 2.8. RNA Extraction and Gene Expression Analysis

Total RNA of xenograft tumors was purified using TRIzol (Thermo Fisher Scientific, Waltham, MA, USA) and, following genomic DNA elimination, 1 µg of RNA was retrotranscribed and analyzed with a PrimePCR custom panel (Bio-Rad, Hercules, CA, USA) (Table 3). *GAPDH*-normalized data were analyzed using the comparative CT method (2^−ΔCt^ method).

### 2.9. Immunofluorescence/Immunohistochemistry

CD44v6/Myc double-staining was performed on 5-µm-thick paraffin-embedded xenograft sections. Antigen retrieval was performed using the PT link system (Dako, Agilent technologies, Santa Clara, CA, USA) with a 10 mM sodium citrate solution (pH 6.0). Before primary antibody incubation, we performed two consecutive blocking steps: one for 5 min with a 3% H_2_O_2_ solution to inhibit endogenous peroxidase and the other for 20 min with 10% human serum to reduce unspecific signals. Thereafter, sections were exposed to antibodies specific for CD44v6 (2F10, R&D systems, Minneapolis, MN, USA) and Myc (rabbit polyclonal, CST, Danvers, MA, USA), diluted in antibody diluent solution (Dako, Agilent technologies, Santa Clara, CA, USA). The MACH 2 Double Stain 2 kit (Biocare Medical, Pacheco, CA, USA) was used to reveal primary antibodies, using DAB and Vulcan Fast Red chromogens for detection. Aqueous hematoxylin (Sigma-Aldrich, St. Louis, MO, USA) was used to counterstain cell nuclei.

CR-CSphCs exposed to vemurafenib, trastuzumab and BKM120 were fixed, permeabilized and incubated overnight at 4 °C with CD44v6 and Myc antibodies. Then, cells were stained with Alexa Fluor-488 goat anti-rabbit IgG and Rhodamine Red-x goat anti-mouse IgG1 (Life Technologies, Waltham, MA, USA) secondary antibodies. Toto-3 Iodide (Life Technologies, Waltham, MA, USA) was used to counterstain nuclei.

### 2.10. In Vivo Experiments

Six- to eight-week-old NOD/SCID mice (Charles River Laboratories, Wilmington, MA, USA) were used for all the in vivo procedures, which were performed following institutional experimental procedure guidelines (Italian Ministry of Health authorization number 154/2017-PR). A total of 2.5 × 10^5^ CR-CSphCs, resuspended in 150 µL of 1:1 SCM/Matrigel (BD) solution, were subcutaneously injected into the flanks of mice. When tumor xenografts reached a volume of 0.03–0.06 cm^3^, mice were randomly divided into control and treatment groups (six mice/group) and treated for 4 weeks with a vehicle, vemurafenib (20 mg/Kg, twice daily, oral gavage), trastuzumab (5 mg/Kg, weekly, intraperitoneal injection (i.p.)), cetuximab (40 mg/Kg, twice weekly, i.p.), BKM120 (20 mg/Kg, daily, oral gavage), taselisib (5 mg/Kg, daily, oral gavage) and dinaciclib (25 mg/Kg, 3 days/week, i.p.).

To evaluate secondary tumor formation, 1 × 10^4^ sphere cells, freshly isolated from treated primary tumor xenografts, were subcutaneously injected into secondary mice recipients (n = 5). Tumor volumes were calculated as follows: largest diameter × (smallest diameter)^2^ × π/6. Mice were sacrificed when tumor xenografts reached 2 cm in tumor diameter or when signs of distress were identified, following Directive 2010/63/EU guidelines (D.lgs 26/2016).

### 2.11. Statistical Analysis

All the results show means ± SD from independent experiments. The means and SD were obtained by analyzing replicates using Prism 5 (GraphPad Software, La Jolla, CA, USA) and applying Student *t*-test. *p*-values greater than 0.05 were considered statistically not significant.

## 3. Results

### 3.1. CD44v6-Positive CR-CSphCs Are Refractory toward BRAF Inhibition

**Figure 1:**
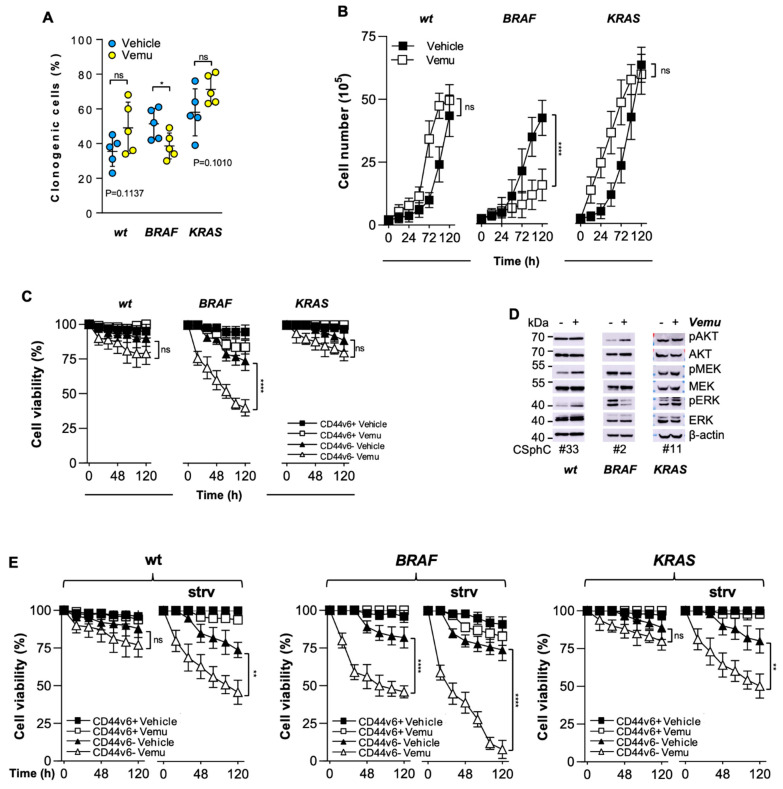
The resistance of CD44v6-positive CR-CSCs to BRAF inhibition is mediated by EGFR-driven MAPK pathway activation. (**A**) Clonogenic assays for wt (#6, #14, #21, #27, #33), *BRAF*- (#1, #2, #3, #4, #5) and *KRAS*-mutant (#9, #10, #11, #13, #16) CSphCs pretreated with a vehicle (Vehicle) or vemurafenib (Vemu, 1 µM) for 120 h. The statistical significance between two groups was determined with a paired two-tailed Student’s *t*-test. (**B**) Kinetics of wt (#14, 21), *BRAF*- (#2, 5) and *KRAS*-mutant (#11, 16) sphere cell growth following treatment with vemurafenib (Vemu, 1 µM) for up to 120 h. (**C**) Cell viability percentage of CD44v6^+^ and CD44v6^−^ wt (#21, #27, #33), *BRAF*- (#1, #3, #5) and *KRAS*-mutant (#9, #13, #16) sphere cells treated as in (**B**). (**D**) Immunoblot analysis of pAKT, AKT, pMEK, MEK, pERK and ERK on the indicated CR-CSphCs treated with a vehicle (−) or vemurafenib (Vemu, +, 1 µM) for 48 h. (**E**) Cell viability percentage of CD44v6^+^ and CD44v6^−^ wt (#21, #27, #33), *BRAF*- (#1, #3, #5) and *KRAS*-mutant (#9, #13, #16) sphere cells treated as indicated in the presence or absence (strv) of EGF for up to 120 h. For (**B**–**C**,**E**), data are means ± SD from three independent experiments. ns, not significant; * *p* ≤ 0.05; ** *p* ≤ 0.01, **** *p* ≤ 0.0001. The uncropped blots are shown in supplementary materials.

**Figure 2:**
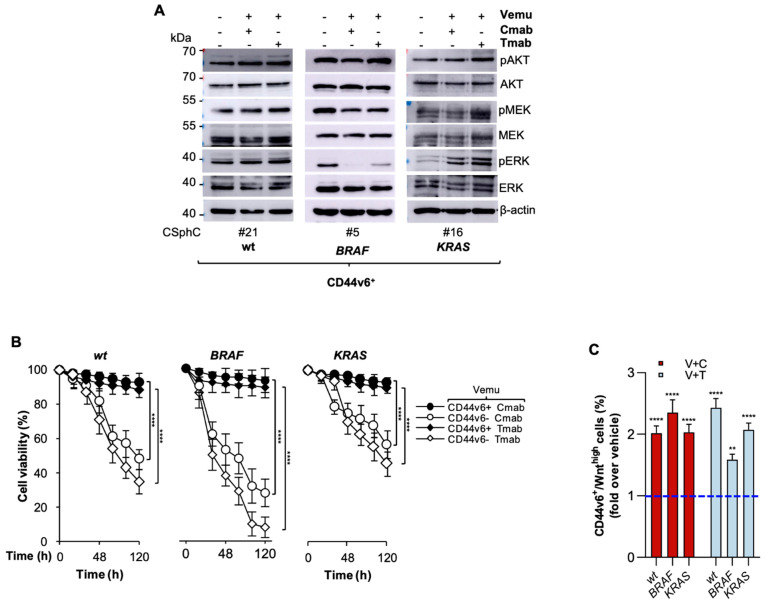
PI3K activation protects CD44v6-positive cells from EGFR/HER2 blockade in combination with BRAF inhibition. (**A**) Immunoblot of pAKT, AKT, pMEK, MEK, pERK and ERK on the indicated CD44v6^+^ cells exposed to a vehicle (−) or to vemurafenib (Vemu, +, 1 µM) in combination with cetuximab (Cmab, +, 20 µg/mL) or trastuzumab (Tmab, +, 10 µg/mL) for 2 h. (**B**) Cell viability percentage in CD44v6^+^ and CD44v6^−^ wt (#21, #27), *BRAF*- (#1, #5) and *KRAS*-mutant (#11, #16) CR-CSphCs treated with vemurafenib (Vemu, 1 µM) in combination with cetuximab (Cmab, 20 µg/mL) or trastuzumab (Tmab, 10 µg/mL). Data are shown as means ± SD of three independent experiments for each CR-CSphC line. (**C**) Fold increase over vehicle (blue dotted line) of CD44v6^+^/Wnt^high^ subpopulation in wt (#27), *BRAF*- (#1) and *KRAS*-mutant (#9) TOP-GFP transduced sphere cells, treated as indicated for 120 h. Data represent the means ± SD of three independent experiments. ** *p* ≤ 0.01, **** *p* ≤ 0.0001.The uncropped blots are shown in supplementary materials.

While treatment with an inhibitor of BRAF (vemurafenib) reduced the clonogenic activity and delayed in vitro growth of *BRAF*-mutant CR-CSphCs, the clonogenicity of *RAS/BRAF*-wt (wt) and *KRAS*-mutant cells showed a trend towards an increase, without altering the proliferation rate (Figure 1A,B and Table 1). Exposure to vemurafenib resulted in minimal or no impact on survival of wt, *BRAF*- and *KRAS*-mutant CD44v6^+^ cells, whereas the CD44v6^−^ fraction of *BRAF*-mutant cells was significantly sensitive to vemurafenib-induced cell death (Figure 1C and Figure S1A). The exposure to different doses of vemurafenib promoted the activation of RAF signaling in wt and *KRAS*-mutant CR-CSphCs, leading to the paradoxical activation of ERK (Figure S1B) [34,35]. Alternatively, following exposure to vemurafenib, *BRAF*-mutant sphere cells attenuated their activation of RAF and strengthened PI3K/AKT signaling (Figure 1D and Figure S1C). Tumorigenic and metastatic CD44v6^+^ populations express higher HER2 than EGFR or HER3 levels [17]. Given that the refractoriness of *BRAF*-mutant CD44v6^+^ cells toward the BRAF inhibitor could be sustained by the activation of RAS and ERK, triggered by EGFR [36], we explored whether EGF deprivation could sensitize this cell compartment to vemurafenib. CD44v6^+^ cells were found to be inherently more resistant to vemurafenib than the CD44v6^−^ cell fraction, even in the absence of EGF and regardless of their mutational profile (Figure 1E). We also evaluated whether the combinatorial targeting of BRAF and EGFR signaling could affect the cell viability of this cell compartment. We used cetuximab, a chimeric human/mouse monoclonal antibody, and trastuzumab, a humanized monoclonal antibody, to target EGFR and HER2, respectively. BRAF/EGFR andHER2 targeting showed similar effects on the MAPK pathway compared with those observed with the use of vemurafenib monotherapy (Figure 2A and Figure S2). Notably, EGFR-based therapy efficiently killed CD44v6^−^ cells, with a more pronounced effect against the *BRAF^V600E^* background. In contrast, CD44v6^+^ cells were largely spared by this dual targeting, which heightened the CD44v6^+^/Wnt^high^ fraction (Figure 2B,C).

### 3.2. CR-CSCs Emerge after Inhibition of HER2, BRAF and PI3K

**Figure 3:**
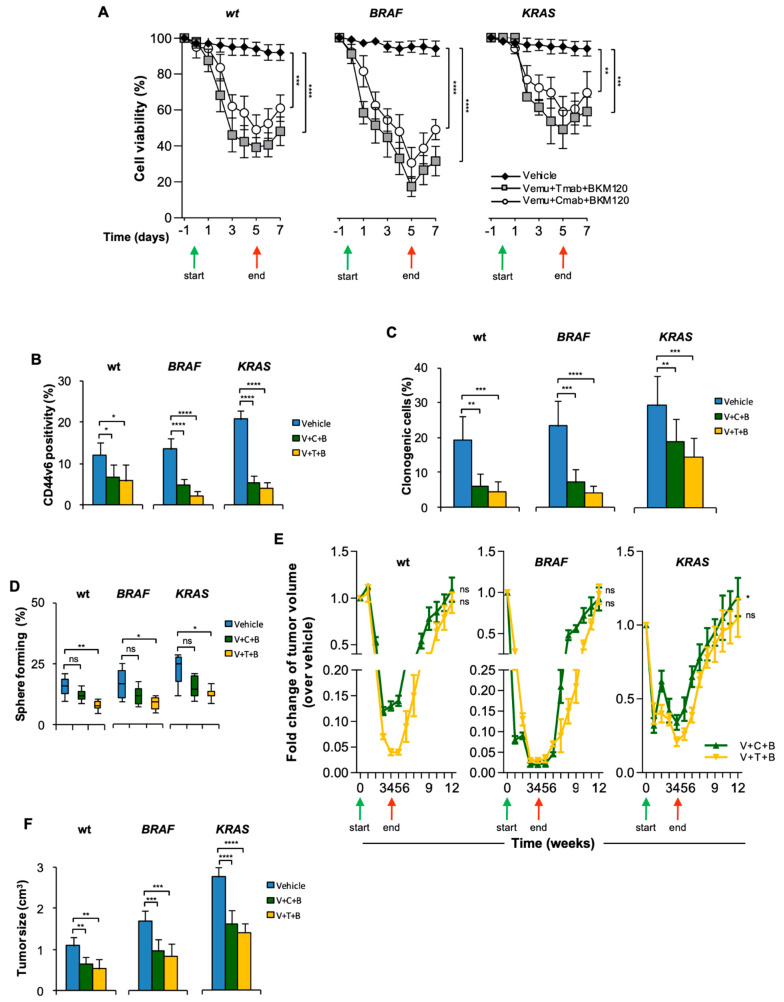
HER2/BRAF/PI3K combinatorial targeting leads to transient therapeutic response. (**A**) Cell viability in wt (#21, #27, #33), *BRAF*- (#1, #3, #5) and *KRAS*-mutant (# 9, #11, #16) CR-CSphCs treated with Vemu (1 µM) + Tmab (10 µg/mL) + BKM120 (1 µM), Vemu + Cmab (20 µg/mL) + BKM120 or with a vehicle as control. (**B**) Percentage of CD44v6 positivity evaluated by flow cytometry for wt (#21, #27, #33), *BRAF*- (#1, #3, #5) and *KRAS*-mutant (#9, #11, #16) CR-CSphCs treated as indicated for 72 h. (**C**,**D**) Clonogenic activity (**C**) and percentage of sphere-forming cells for wt (#21, #27, #33), *BRAF*- (#1, #3, #5) and *KRAS*-mutant (#9, #11, #16) sphere cells pretreated as in (**B**). For (**A**–**D**), data show the means ± SD of three different experiments. (**E**) Fold change over vehicle of volume of subcutaneous tumor xenograft generated by the injection of wt (#14, 21), *BRAF*- (#1, 2) or *KRAS*-mutant (#11, 16) CR-CSphCs and treated with a vehicle (Vehicle), vemurafenib (V, 20 mg/Kg), cetuximab (C, 40 mg/Kg) or trastuzumab (T, 5 mg/Kg) in combination with BKM120 (B, 20 mg/Kg) for 4 weeks. For (**A**,**E**), green and red arrows indicate the start and the end of the treatment, respectively. (**F**) Size of secondary tumors (8 weeks) generated by subcutaneous injection of 1 × 10^4^ freshly purified CR-CSphCs derived from wt (#21), *BRAF*- (#2) or *KRAS*-mutant (#11) xenograft tumors. For (**D**,**E**), data are expressed as means ± SD (n = 6). ns, not significant; * *p* ≤ 0.05, ** *p* ≤ 0.01, *** *p* ≤ 0.001, **** *p* ≤ 0.0001.

Given that CD44v6^+^ cells display enhanced activation of PI3K/AKT signaling that in turn sustains the β-catenin activation [11,37], BKM120, an inhibitor of class I isoforms of PI3K, was supplemented to the anti-EGFR/HER2 and BRAF inhibitor combination. The triple combination transiently decreased the in vitro viability of CR-CSphCs, which restarted growth after treatment suspension (Figure 3A). CR-CSphCs, exposed to this treatment displayed a depletion of CD44v6^+^ cells together with reduced clonogenic and sphere-forming activity, suggesting that CD44v6^+^ cells are considerably affected by the addition of BKM120 (Figure 3B–D). We next evaluated the efficacy of this PI3K inhibitor-based therapy in tumor xenografts. This treatment initially, at 5 weeks, produced a stabilizing effect in the growth of *BRAF*-mutated xenograft tumors and led to a delayed progression of wt and *KRAS*-mutated xenograft tumors. Eight weeks after treatment suspension, xenograft tumors exhibited consistent regrowth (Figure 3E and Figure S3A). At 5 weeks, the CRC cells surviving this combination treatment displayed tumorigenic activity that was able to generate secondary tumors when extracted and re-injected into immunocompromised mice (Figure 3F). Cells harvested from these secondary tumors showed a comparable percentage of clonogenic cells (Figure S3B), suggesting that resistant tumorigenic cells are spared by PI3K-based combinatorial treatments.

### 3.3. Resistance to BRAF-Based Combination Therapy Is Sustained by Myc Expression

**Figure 4:**
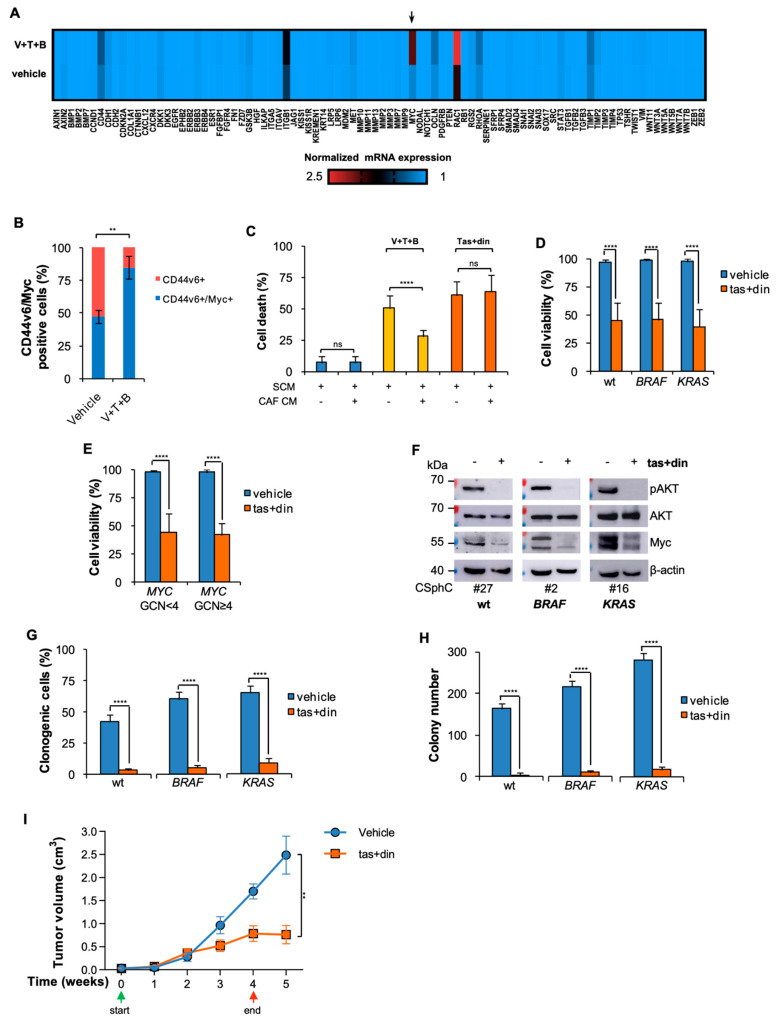
Myc and PI3K combinatorial targeting reduces CR-CSphC viability, downregulating Myc and CD44v6 expression levels. (**A**) Heatmap of cancer stem cell- and metastasis-related genes (2^−ΔCt^) in freshly purified cells (#21, #2, #10) from xenografts treated with a vehicle or V (20 mg/Kg) + T (5 mg/Kg) + B (20 mg/Kg). (**B**) Percentage of CD44v6 and Myc expression in xenograft tumors treated for 4 weeks with a vehicle (Vehicle) or V (20 mg/Kg) + T (5 mg/Kg) + B (20 mg/Kg). Data are shown as means ± SD of three different xenografts (#21, #2, #11). (**C**) Death percentage of CR-CSphCs treated as indicated in the presence of SCM or CAF-conditioned medium (CAF CM) for 72 h. Data represent means ± SD of three independent experiments performed with wt (#21, #27, #33), *BRAF*- (#1, #3, #5) and *KRAS*-mutant (#8, #9, #16) CR-CSphCs. (**D**) Cell viability in wt (#6, #14, #21, #27, #33, #49), *BRAF*- (#1–#5) and *KRAS*-mutant (#8, #9, #11, #13, #16, #59) CR-CSphCs treated with vehicle or taselisib (1 µM) and dinaciclib (10 nM) (tas + din) for 72 h. (**E**) Cell viability in *MYC* wt (*MYC* GCN < 4, CSphC#2, #3, #6, #9, #14, #16, #21, #27, #33) and *MYC*-amplified (*MYC* GCN ≥ 4, #1, #4, #5, #8, #11, #13, #49, #59) CR-CSphCs treated as in (**D**). For (**C**,**D**), data represent means ± SD of three independent experiments. (**F**) Analysis of pAKT, AKT and Myc on the indicated CR-CSphCs and treated with a vehicle or tas (1 µM) + din (10 nM) at 48 h. (**G**,**H**) Clonogenic activity (**G**) and colony number (**H**) of wt (#21, #33), *BRAF*- (#1, #3) and *KRAS*-mutant (#9, #16) sphere cells previously treated with a vehicle or tas (1 µM) + din (10 nM) for 72 h, in the presence of CAF CM. For (**G**,**H**), data are means ± SD of three independent experiments. For (**D**–**H**), CR-CSphCs were exposed to CAF CM. (**I**) Tumor volume of subcutaneous xenograft generated by the injection of *KRAS*-mutant (#8) CR-CSphCs and treated with a vehicle (Vehicle) or taselisib (tas, 5 mg/Kg) and dinaciclib (din, 25 mg/Kg) for 4 weeks. Green and red arrows indicate the start and the end of the treatment, respectively. Data are expressed as means ± SD (n = 6). ns, not significant; ** *p* ≤ 0.01, **** *p* ≤ 0.0001. The uncropped blots are shown in supplementary materials.

To investigate the molecular mechanisms underlying the triple-combination acquired resistance, cancer cells isolated from tumor xenografts were subjected to gene expression analysis of cancer stem cell- and metastasis-related genes. Notwithstanding the fact that the expression levels of genes involved in the Wnt pathway and EMT process were not influenced, cancer cells surviving the in vivo combinatorial treatment showed a markedly increased expression of *MYC* levels, regardless of the mutational status (Figure 4A). This expression was mainly restricted to CD44v6^+^ cells surviving the triple combination (Figure 4B and Figure S4A). Although Myc expression levels are altered in many tumors and numerous therapeutic strategies to directly target them have been evaluated, Myc still remains “undruggable” [38]. To study whether the inhibition of CD44v6 and Myc could efficiently target CR-CSCs, we combined dinaciclib, a selective small-molecule inhibitor of CDKs that indirectly downregulates Myc [39], with taselisib, a selective inhibitor of class I PI3K currently used in clinical settings. CR-CSphCs were exposed to taselisib in combination with dinaciclib in a dose–response matrix (Figure S4B). Thus, we sought to investigate whether microenvironmental cytokines, released by cancer-associated fibroblasts (CAFs), would affect the viability of CR-CSphCs treated with dinaciclib-based therapy, which targets downstream of the MEK pathway. In accordance with our previously published data [17], CAF-conditioned medium (CAF CM) played a protective effect in CR-CSphCs exposed to vemurafenib-based therapy, increasing the CD44v6^+^ cell population expressing Myc (Figure 4C and Figure S4C). This phenomenon was abrogated when using Myc and PI3K inhibitors in CR-CSphCs, the survival of which was significantly reduced regardless of the mutational status (Figure 4C,D). In the presence of CAF CM, dinaciclib in combination with taselisib reduced the cell viability of CR-CSphCs harboring *MYC* amplification and lowered the activity of the PI3K/AKT pathway and Myc expression (Figure 4E,F and Figure S4D). Notably, this treatment was able to effectively counteract clonogenic and colony-forming activities (Figure 4G,H and Figure S4E). Targeting of Myc transcription and CD44v6 with dinaciclib and taselisib, respectively, significantly affected the growth of tumor xenografts generated by subcutaneous injection of aggressive *KRAS*-mutant/*MYC*-amplified CR-CSphCs, without affecting mice weight (Figure 4I and Figure S4F). Altogether, these findings indicate that Myc promotes CSC resistance to BRAF-based combination therapy and its indirect inhibition overwhelms the protective activity of the TME, paving the way for an investigation of dinaciclib in combination with taselisib in clinical regimens.

## 4. Discussion

One of the major challenges in cancer research is preventing the emergence of acquired resistance to targeted therapies. Thus, the discovery of targetable resistance mechanisms may allow the design of new therapeutic strategies to overcome tumor recurrence.

The most frequent deregulated pathways in CRC patients are the Wnt, MAPK and PI3K pathways [40]. Patients harboring *RAS/RAF* mutations display poor prognosis, highlighting a critical need to improve therapeutic options [41]. CRC patients treated with selective Ras/Raf pathway-targeted agents show limited benefits in contrast to the considerable response observed in *BRAF*-mutated melanoma patients [2]. Acquired drug resistance to this combination therapy is mediated by feedback reactivation of EGFR via the Ras pathway, triggering cell proliferation and survival [36,42]. Although a clinical advantage was observed in *BRAF*-mutant CRC patients treated with BRAF, EGFR and MEK inhibitors, the extent of the clinical responses was time-limited, suggesting the urgency of discovering molecular mechanisms driving CRC acquired resistance [43].

As the data show, the vemurafenib treatment produced a short-term inhibition of colony-forming capacity and cell viability in *BRAF*-mutant CR-CSphCs due to EGFR-mediated activation of ERK signaling. Moreover, a paradoxical activation of the ERK pathway was observed in wt and *KRAS*-mutant cells, corroborating the finding that the aberrant activity of EGFR signaling may contribute to conferring resistance to vemurafenib in CRC cells.

We recently identified CD44v6 as a functional stem cell-like marker that typifies CR-CSCs characterized by metastatic potential and standard/targeted therapy resistance [11,17,30,44,45]. Independently of their mutational background, CD44v6-negative and -positive fractions displayed different sensitivities to BRAF inhibition in combination with anti-EGFR/HER2 antibodies, limiting their efficacy to the more differentiated cells. This CD44v6^+^ cell refractoriness is sustained by high expression levels of HER2, associated with an aberrant activation of PI3K-AKT and MAPK signaling [17]. Although PI3K and AKT inhibitors efficiently target disseminating CD44v6^+^ CR-CSCs, their anti-tumor activity was found to be dramatically reduced in pre-clinical mouse models, where the presence of a protective TME counteracted drug efficacy [11,17]. Likewise, cells spared by BRAF, HER2 and PI3K inhibition maintained the capacity to generate secondary tumors and re-grow following conclusion of treatment, even though reductions of CD44v6 expression and sphere-forming ability were observed in primary tumor xenograft-derived cells [17]. A gene expression analysis of cancer cells exposed to that treatment identified Myc and Rac1, one of the critical factors in the maintenance of the intestinal barrier and upregulated in primary and metastatic CRCs [46], as major candidates driving acquired resistance. This phenomenon could be driven by the adaptive responses to PI3K-AKT signaling pathway inhibition, which leads to Myc induction by activating NOTCH signaling, which is a hallmark of CSCs [47]. Although a good candidate for the development of therapeutic targeted strategies, pharmacological Myc inhibition remains difficult since both its nuclear localization and biological function are critical for tissue homeostasis. JQ1 is a small-molecule inhibitor of BRD4, a member of the bromodomain and extraterminal (BET) family regulating the transcription of *MYC*. Although JQ1 induces cell death by affecting the growth of several cancers, its bioavailability and short half-life hinder its use in clinical settings [48,49]. Several clinical trials have been designed with analogs of JQ1, such as AZD5153 and GSK525762, to indirectly target Myc, both in hematological and solid neoplasia (i.e., NCT03205176, NCT01587703). Despite their potent anti-tumor activity, BET inhibitors in patients displayed a high toxicity profile [50,51]. Another strategy adopted to interfere with *MYC* transcription is the use of cyclin-dependent kinase (CDK) inhibitors [50]. A clinical trial involving dinaciclib, a potent multiple CDK inhibitor, in advanced breast cancer patients is currently evaluating the response based on Myc expression (NCT01676753) [51]. Additionally, Myc-dominant negative mutant Omomyc has been recently approved for its first human phase I/III trial to assess its efficacy in cancer patients [52]. Moreover, amplification of *MYC* has been reported as a key biomarker of response to HER2 inhibitors [53]. The synthetic lethality approach based on dinaciclib in combination with taselisib impairs CR-CSphCs viability and clonogenic capacity, with a concomitant reduction of Myc and PI3K/AKT pathway activity in the presence of TME cytokines, in line with the stabilization of tumor growth in mouse avatars. The possibility of interfering with *MYC* transcription, a downstream target of MAPK pathway, could provide potential clinical benefits by reducing the side effects of kinase inhibitors and preventing acquired resistance mediated by ERK reactivation. *RAS* is the most frequently mutated oncogene in cancer and about the 45% of CRCs harbor *KRAS* mutations. Notably, targeting of Rac1 by depletion of specific Rac-guanine nucleotide exchange factors (Rac-GEFs) fails to hinder the tumor growth in the presence of *KRAS* mutations, limiting its clinical use for advanced tumors [54].

Despite the efforts that have been made toward the development of efficacious therapeutic regimens, to date no targeted therapies are available for these CRC patients. Recently, several clinical trials have evaluated the anti-tumor activities of small molecules (AMG510 and MRTX84) targeting mutant *KRAS^G12C^* mutation (NCT03600883, NCT03785249). Adaptive feedback signaling networks that lead to activation of the EGFR pathway after *KRAS^G12C^* inhibitors were recently identified as the dominant mechanism of resistance in CRC patients [55].

Here, we identified a potential mechanism, shared by CRCs, of acquired resistance to targeted cancer therapies. Under drug treatment pressure, the presence of TME cytokines promotes CD44v6 expression together with high expression levels of Myc, representing an escape mechanism adopted by cancer cells to cope with the action of anti-tumor therapies and favor disease progression. Taken together, these data provide a strong rationale for the use of dinaciclib combined with taselisib for advanced CRCs.

## 5. Conclusions

Herein, we demonstrated that the combinatorial targeting of *MYC* transcription and PI3K activity could be a valuable therapeutic option for advanced CRC, regardless of the mutational background and/or *MYC* gene amplification. We found that the combinatorial targeting of BRAF and EGFR/HER2 spares the CD44v6-positive subpopulation endowed with high PI3K/AKT activity. Although adding a PI3K inhibitor stabilized/delayed the progression of xenograft tumors, following treatment cessation, the tumor microenvironment boosted the expression of CD44v6 and Myc, resulting in tumor xenograft regrowth. The targeting of *MYC* transcription and PI3K activity impaired CR-CSphCs’ viability and clonogenic capacity, even in the presence of microenvironmental cytokines, and stabilized tumor xenograft growth, supporting the use of this combinatorial treatment for advanced CRC patient management.

## Figures and Tables

**Table 1:** CD44v6 expression levels, *MYC* copy number variation, MSI status and *BRAF/KRAS* mutational status of colorectal cancer sphere cells (CR-CSphCs).

CD44v6 expression																																						
*MYC* GCN																																						
MSI status																																	NA					
Site	S	L	R	R	R	R	R	R	S	L	R	R	R	R	L	R	L	S	R	R	M	M	R	NA	L	R	R	R	R	S	S	R	S	R	S	L	L	M
*BRAF*																																						
*KRAS*																																						
CSphC #	1	2	3	4	5	6	7	8	9	10	11	12	13	14	15	16	17	19	20	21	22	23	24	25	27	29	30	33	37	44	49	52	53	55	56	57	58	59
**CD44v6 expression levels (%)**															***MYC/EIF2C1* ratio**										**MSI status**									**Mutation Type**				
high												>70%			*MYC* GCN < 4										stable									wt				
medium												30–70%			*MYC* GCN ≥ 4										low									Missense				
low												<30%													high													
NA																																						

**Table 2:**
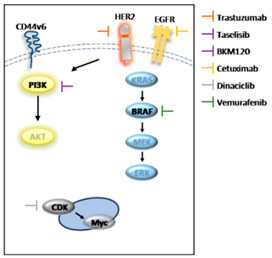
List and diagram illustrating the site of action, type and target of each drug.

Name	Type	Target
Vemurafenib	Small molecule	*BRAF* ^V600E^
Cetuximab	Recombinant human/mouse chimeric IgG(1) monoclonal antibody	EGFR
Trastuzumab	Recombinant humanized monoclonal antibody	HER2
BKM120	dimorpholino pyrimidine derivative	class I PI3K
Dinaciclib	small molecule	CDK1/2/5/9
Taselisib	small molecule	*PIK3CA*

**Table 3:** List of cancer stem cell- and metastasis-related genes.

Gene Name	Assay ID	Gene Name	Assay ID	Gene Name	Assay ID
AXIN1	qHsaCID0010131	ILKAP	qHsaCID0011777	SERPINE1	qHsaCID0006432
AXIN2	qHsaCID0017930	ITGA5	qHsaCID0021495	SFRP1	qHsaCID0015548
BMP1	qHsaCID0010875	ITGAV	qHsaCID0006233	SFRP4	qHsaCED0043151
BMP2	qHsaCID0015400	ITGB1	qHsaCED0005248	SMAD2	qHsaCID0022031
BMP7	qHsaCID0011038	JAG1	qHsaCID0006831	SMAD4	qHsaCID0015670
CCND1	qHsaCID0013833	KISS1	qHsaCED0004976	SNAI1	qHsaCED0002998
CD44	qHsaCID0013679	KISS1R	qHsaCID0011504	SNAI2	qHsaCID0011342
CDH1	qHsaCID0015365	KREMEN1	qHsaCED0047626	SNAI3	qHsaCED0047761
CDH2	qHsaCID0015189	KRT14	qHsaCED0047868	SOX17	qHsaCED0001884
CDKN2A	qHsaCED0023006	LRP5	qHsaCED0045974	SRC	qHsaCED0004489
COL1A1	qHsaCED0002181	LRP6	qHsaCID0010231	STAT3	qHsaCID0010912
CTNNB1	qHsaCID0010363	MDM2	qHsaCID0011000	TGFB1	qHsaCID0017026
CXCL12	qHsaCID0012398	MET	qHsaCED0002004	TGFB2	qHsaCID0018360
CXCR4	qHsaCED0002020	MMP2	qHsaCID0015623	TGFB3	qHsaCID0022239
DKK1	qHsaCED0002060	MMP3	qHsaCID0006170	TIMP1	qHsaCID0007434
DKK3	qHsaCED0001115	MMP7	qHsaCID0011537	TIMP2	qHsaCID0022953
EGFR	qHsaCID0007564	MMP9	qHsaCID0011597	TIMP3	qHsaCID0015238
EPHB2	qHsaCID0009881	MMP10	qHsaCED0046399	TIMP4	qHsaCID0016129
ERBB2	qHsaCID0012766	MMP11	qHsaCID0022136	TP53	qHsaCID0013658
ERBB3	qHsaCID0018397	MMP13	qHsaCID0008487	TSHR	qHsaCID0009606
ERBB4	qHsaCID0017862	MYC	qHsaCID0012921	TWIST1	qHsaCED0003856
ESR1	qHsaCED0033920	NODAL	qHsaCID0006123	VIM	qHsaCID0012604
FGFBP1	qHsaCID0015948	NOTCH1	qHsaCID0011825	WNT3A	qHsaCED0045634
FGFR4	qHsaCED0045915	OCLN	qHsaCED0038290	WNT5A	qHsaCID0012240
FN1	qHsaCID0012349	PDGFRB	qHsaCID0013272	WNT5B	qHsaCID0038673
FZD7	qHsaCED0019290	PTEN	qHsaCED0036796	WNT7A	qHsaCID0018523
GAPDH	qHsaCED0038674	RAC1	qHsaCED0001330	WNT7B	qHsaCED0003528
GSK3B	qHsaCID0010097	RB1	qHsaCID0007095	WNT11	qHsaCID0011927
HGF	qHsaCID0011441	RGS2	qHsaCED0001744	ZEB1	qHsaCID0009210
HPRT1	qHsaCID0016375	RHOA	qHsaCID0008947		

## Data Availability

Data related to the study are included in the article or uploaded as supplementary information. Data are available from the corresponding authors (R.D.M. and G.S.) upon reasonable request.

## Supplementary Materials

The following are available online at https://www.mdpi.com/article/10.3390/cancers14030673/s1, Figure S1: BRAF inhibition leads to paradoxical activation of MAPK pathway in wt and *KRAS*-mutant CR-CSphCs. Figure S2: The addition of EGFR/HER2 inhibitor to vemurafenib results in no additional effect on the MAPK pathway. Figure S3: Xenograft-derived CR-CSphCs following treatment with HER2/BRAF/PI3K inhibition maintain their sphere-forming capacity. Figure S4: Combinatorial inhibition of Myc and PI3K overcome the protective effect of TME cytokines. The uncropped blots are shown in supplementary materials.
