# Supplementary material for: Dual Inhibition of Myc Transcription and PI3K Activity Effectively Targets Colorectal Cancer Stem Cells
Source: Cancers (Basel). 2022 Jan 28;14(3):673. doi: 10.3390/cancers14030673 (PMC8833549; doi:10.3390/cancers14030673)

Figure S1. BRAF inhibition leads to paradoxical activation of MAPK pathway in wt and *KRAS* mutant CR-CSphCs. (A) Representative images of non clonogenic and clonogenic wt (#27), *BRAF*- (#3) and *KRAS*-mutant (#16) CSphCs. Scale bars, 50 µm **(B)** Sorting strategy for CD44v6 negative and positive CR-CSphC fractions. **(C)** Immunoblot analysis and its relative pERK/ERK OD ratio of in wt (CR-CSphC#21, #33), *BRAF*- (CR-CSphC#2, #5), and *KRAS*- (CR-CSphC#11, #16) mutant sphere cells treated with vehicle or the indicated concentration of vemurafenib (Vemu). β-actin was used as a loading control. Data are mean ± SD of 2 independent experiments. **(D)** Relative band densities of pAKT, AKT, pMEK, MEK, pERK, ERK in wt (CR-CSphC#21, #33), BRAF- (CR-CSphC#2, #5) and KRAS- (CR-CSphC#11, #16) cells treated with vehicle and vemurafenib (1 µM) for 48 hours. Data are mean ± SD of 2 independent experiments. ns, no significant; * $p \leq 0.05$, **$p \leq 0.01$, *** $p \leq 0.001$****$p \leq 0.0001$.

Figure S2. The addition of EGFR/HER2 inhibitor to vemurafenib display no additional effect on MAPK pathway. (A) Relative OD ratio analysis of pAKT, AKT, pMEK, MEK, pERK, ERK in wt (CSphC#21, #27) *BRAF*-(CSphC#2, #5) and *KRAS*- (CSphC#9, #16) mutant sphere cells treated with vehicle (-) or with vemurafenib (V, 1 µM) in combination with cetuximab (C, 20 µg/mL) or trastuzumab (T, 10 µg/mL) for 2 hours. Data are mean ± SD of 2 independent experiments. ns, no significant; **$p \leq 0.01$, *** $p \leq 0.001$****$p \leq 0.0001$.

Figure S3. Xenograft-derived CR-CSphCs following treatment with HER2/BRAF/PI3K inhibition maintain the sphere forming capacity. (A) Representative images of spheres obtained from wt (#27), *BRAF*- (#3) and *KRAS*-mutant (#9) CSphCs untreated and treated as indicated. Scale bars, 100 µm **(B)** Survival rate percentage up to 12 weeks of mice (n=5 mice per group) injected with of wt (CSphC#14), *BRAF*- (CSphC#2) or *KRAS*- (CSphC#16) mutant sphere cells and treated as indicated for 4 weeks. Arrows indicate the start and end of treatment. **(C)** Percentage of sphere forming capacity on cells derived from secondary tumors (8 weeks) generated by subcutaneous injection of 10^4 freshly purified CRC cells derived from xenografts bearing the indicated mutations and treated with vehicle (Vehicle) or vemurafenib (Vemu, 20 mg/Kg) in combination with cetuximab (Cmab, 40 mg/Kg) or trastuzumab (Tmab, 5 mg/Kg) plus PI3K inhibitor (BKM120, 20 mg/Kg). Data are expressed as mean ± SD of 3 independent experiments using wt (CSphC#14), *BRAF*- (CSphC#2) or *KRAS*- (CSphC#16) mutant xenograft-derived sphere cells.

Figure S4. Combinatorial inhibition of Myc and PI3K overcome the protective effect of TME cytokines. (A) Representative immunohistochemical analysis of CD44v6 (red) and Myc (brown) on tumor xenografts generated by the injection of *BRAF*-mutant (CR-CSphC#2) Cr-CSphCs treated as indicated. Scale bars, 20 µm. **(B)** Dose response matrix (SynergyFinder (version 2.0)) of wt (CSphC#21), *BRAF*- (CSphC#5) or *KRAS*- (CSphC#16) mutant sphere cells treated with taselisib (1 µM) and dinaciclib (10 nM), alone or in combination, at the indicated doses for 72 hours. **(C)** Representative immunofluorescence analysis of sphere cells treated as indicated, in presence of CAF CM, for 72 hours. Nuclei were counterstained with TOTO-3. (*lower panels*) Cell positivity of CD44v6 and Myc on wt (CSphC#21, #27), *BRAF*-(CSphC#2, #5) and *KRAS*- (CSphC#11, #16) mutant sphere

cells treated with vehicle or the triple combination (V+T+B). Scale bars, 20 µm. Data are expressed as mean ± SD of 3 independent experiments **(D)** Relative band densities of pAKT, AKT and Myc in wt (CR-CSphC#21, #27), *BRAF*- (CR-CSphC#2, #5) and *KRAS*- (CR-CSphC#8, #16) cells treated with vehicle and taselisib (1 µM) in combination with dinaciclib (10 nM) (Tas + Din) for 48 hours. Data are expressed as mean ± SD. **(E)** Representative colony-forming assay of the indicated CR-CSphCs previously treated with vehicle (Vehicle) or taselisib (tas, 1 µM) in combination with dinaciclib (din, 10 nM) for 72 hours, in presence of CAF CM. **(F)** Kinetics of body weight oscillation in mice treated for four weeks as in Figure 4I. Time "0" indicate the start of treatment. Green and red arrows indicate the start and the end of the treatment, respectively. Data are expressed as mean ± SD (n=6). ns, no significant; * $p \leq 0.05$, **$p \leq 0.01$, *** $p \leq 0.001$****$p \leq 0.0001$.

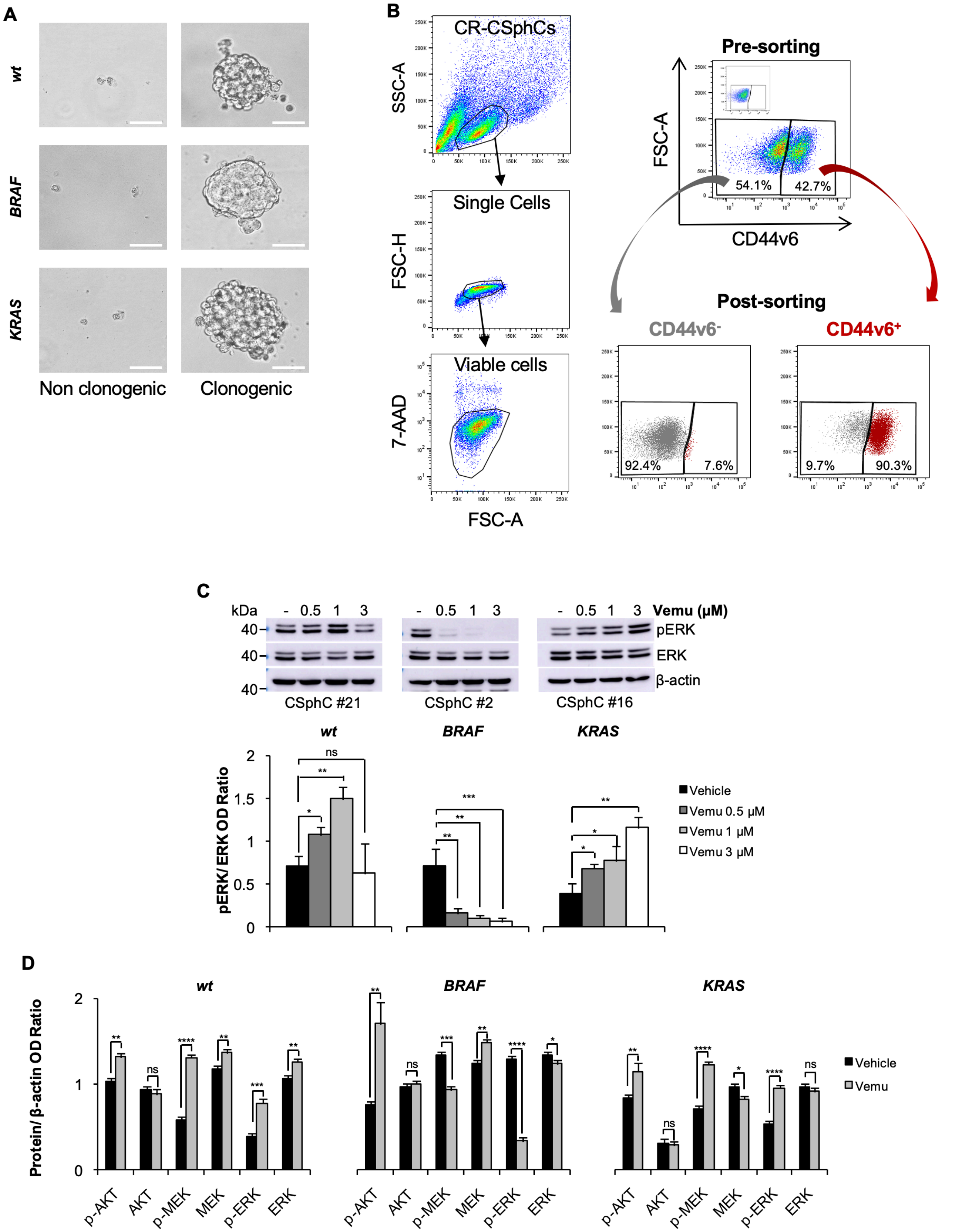

Gaggianesi et al., Figure S1

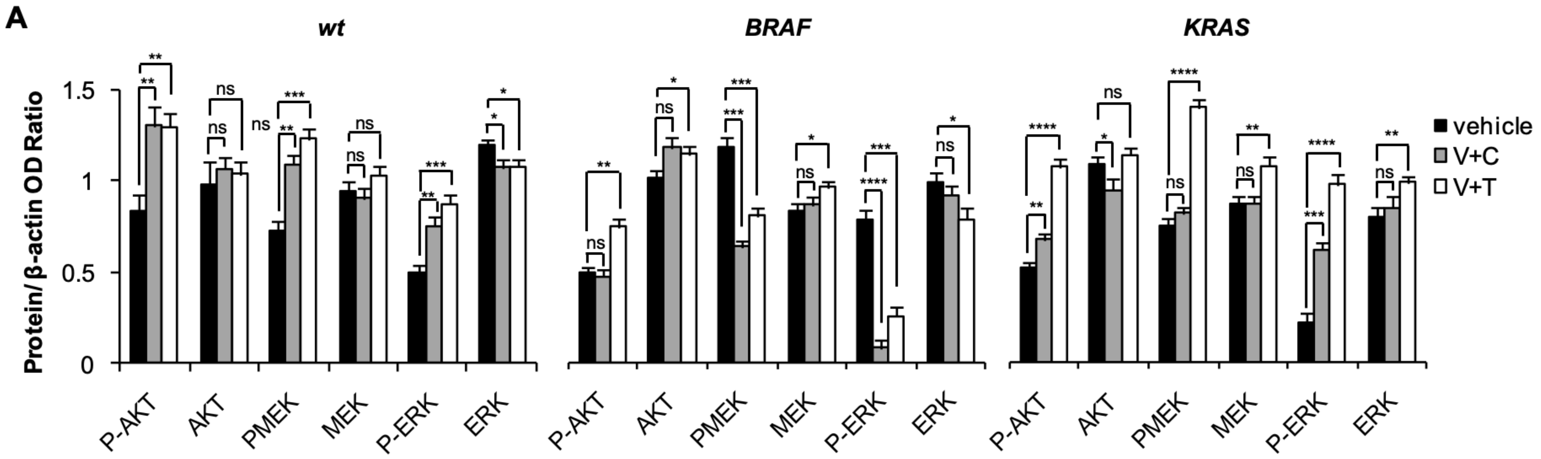

Gaggianesi et al., Figure S2

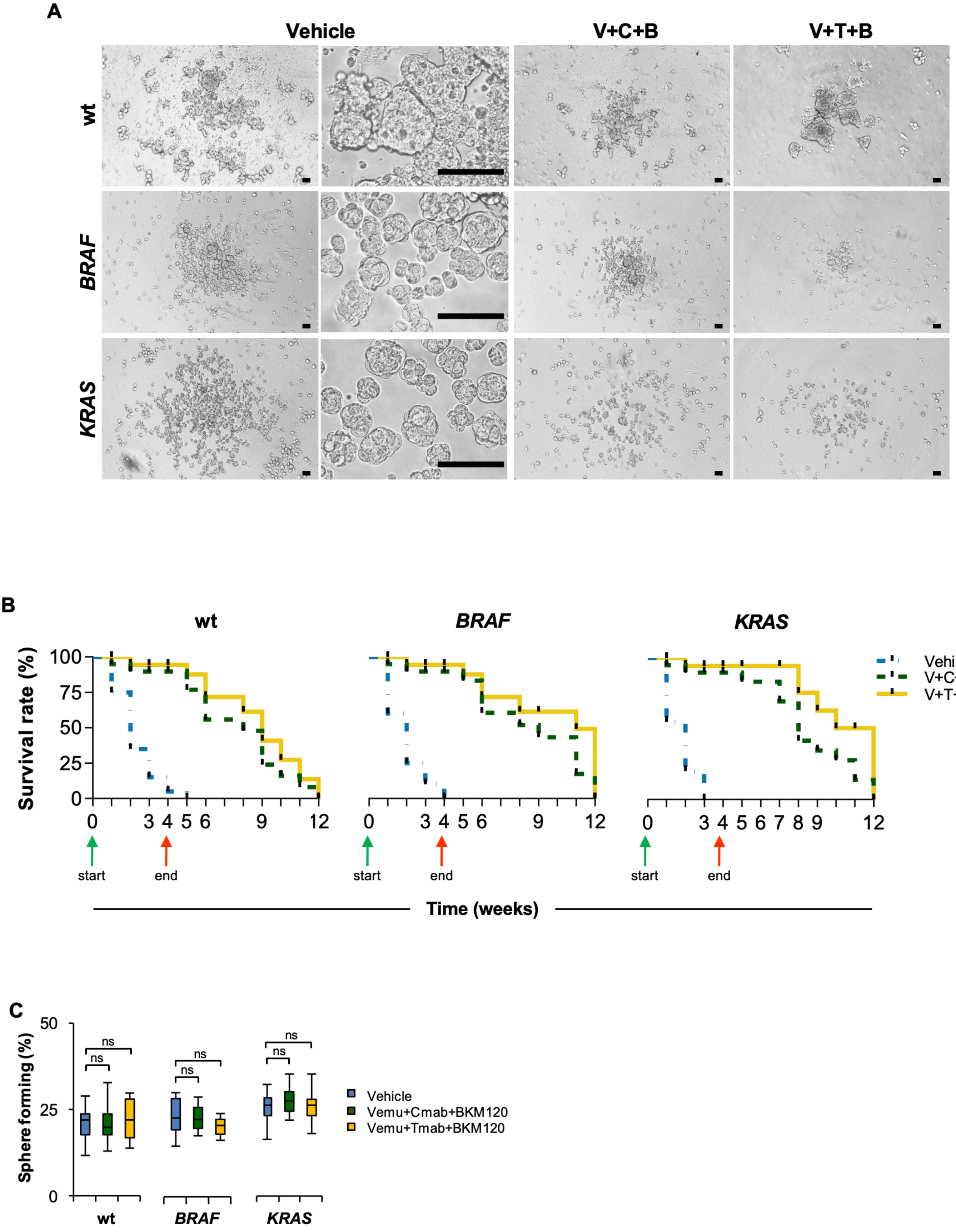

Gaggianesi et al., Figure S3

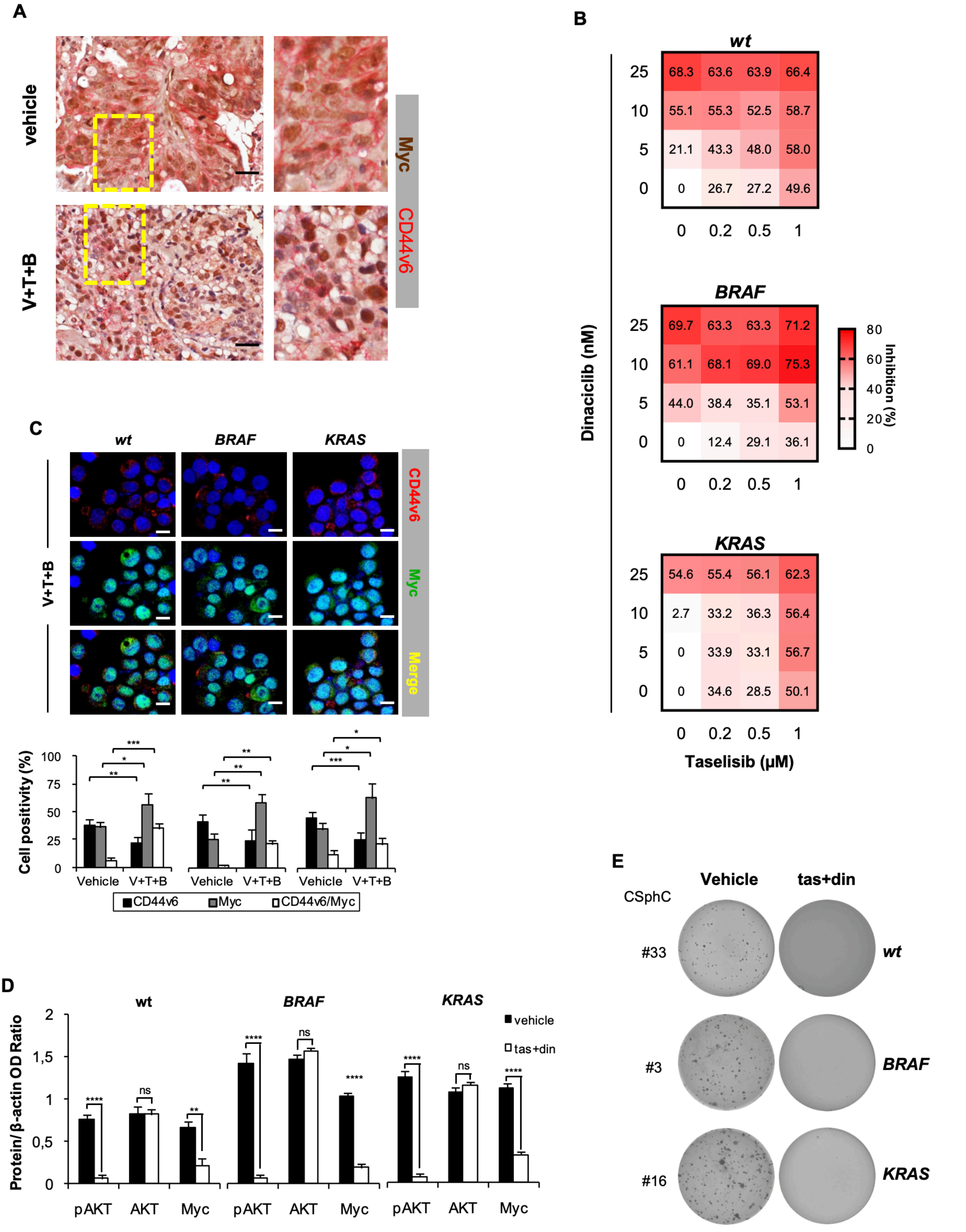

Gaggianesi et al., Figure S4

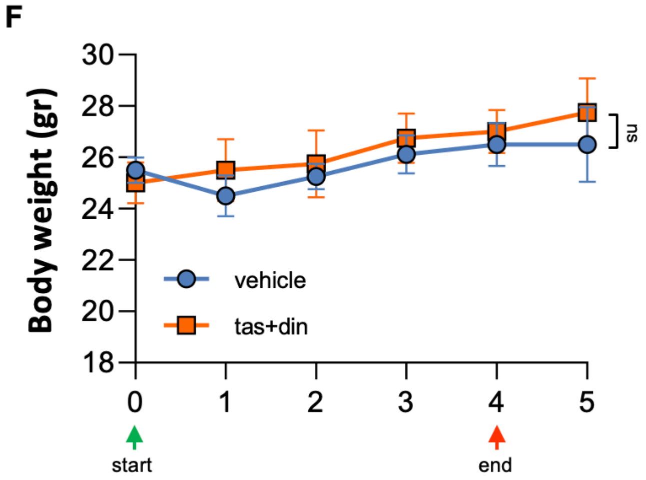

Gaggianesi et al., Figure S4

Full unedited gel for Figure 1D

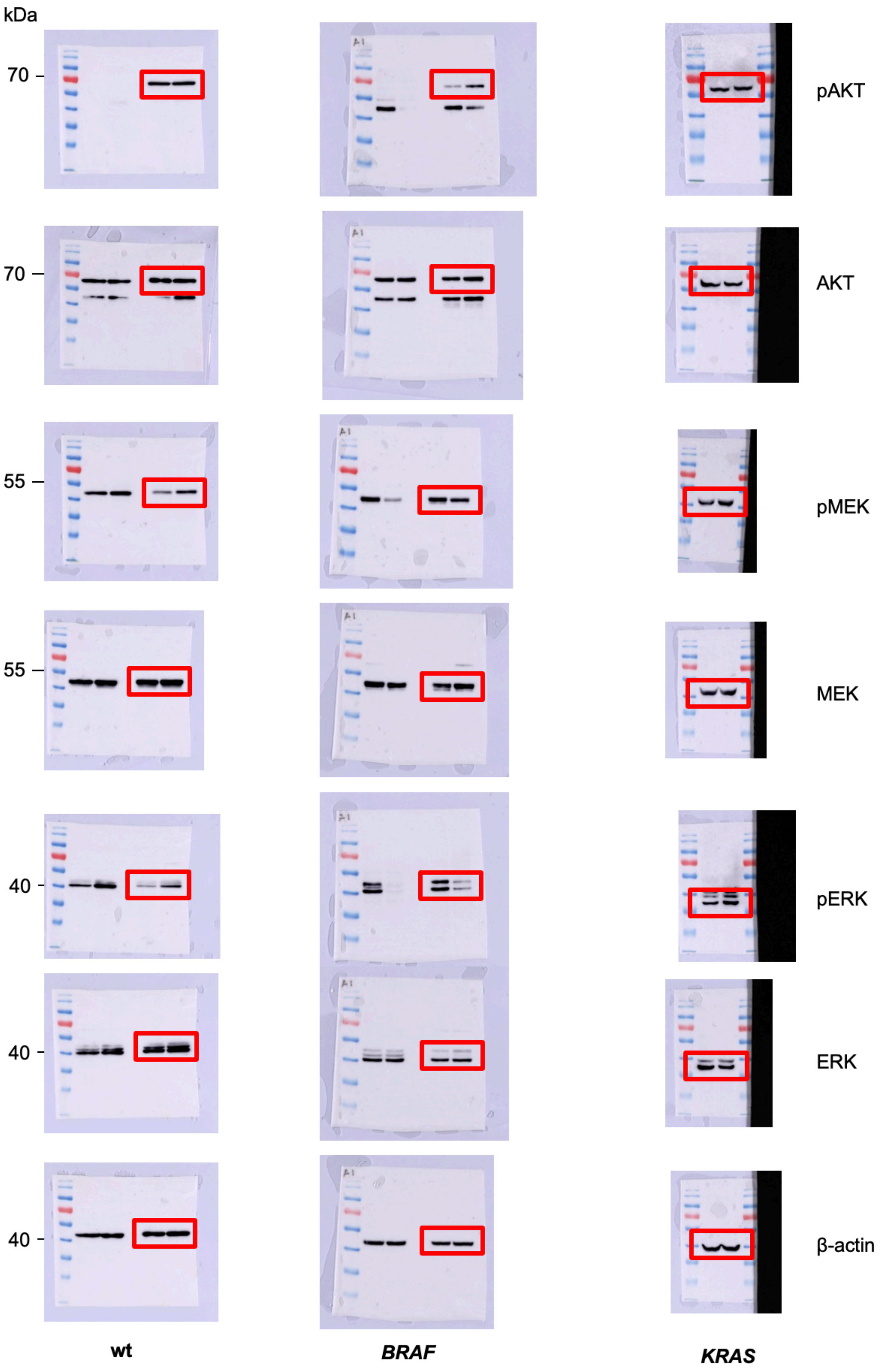

Full unedited gel for Figure 2A

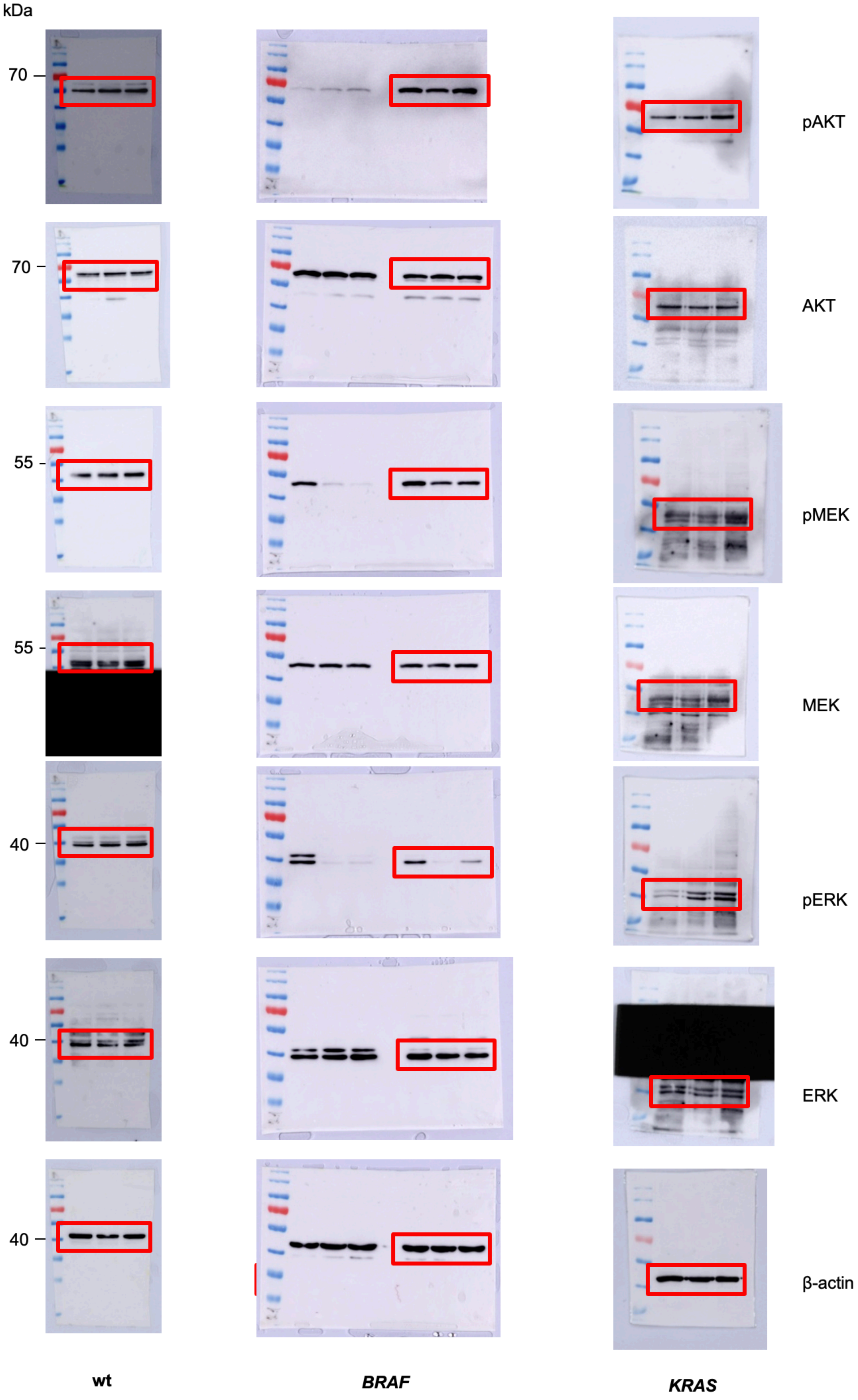

Full unedited gel for Figure 4F

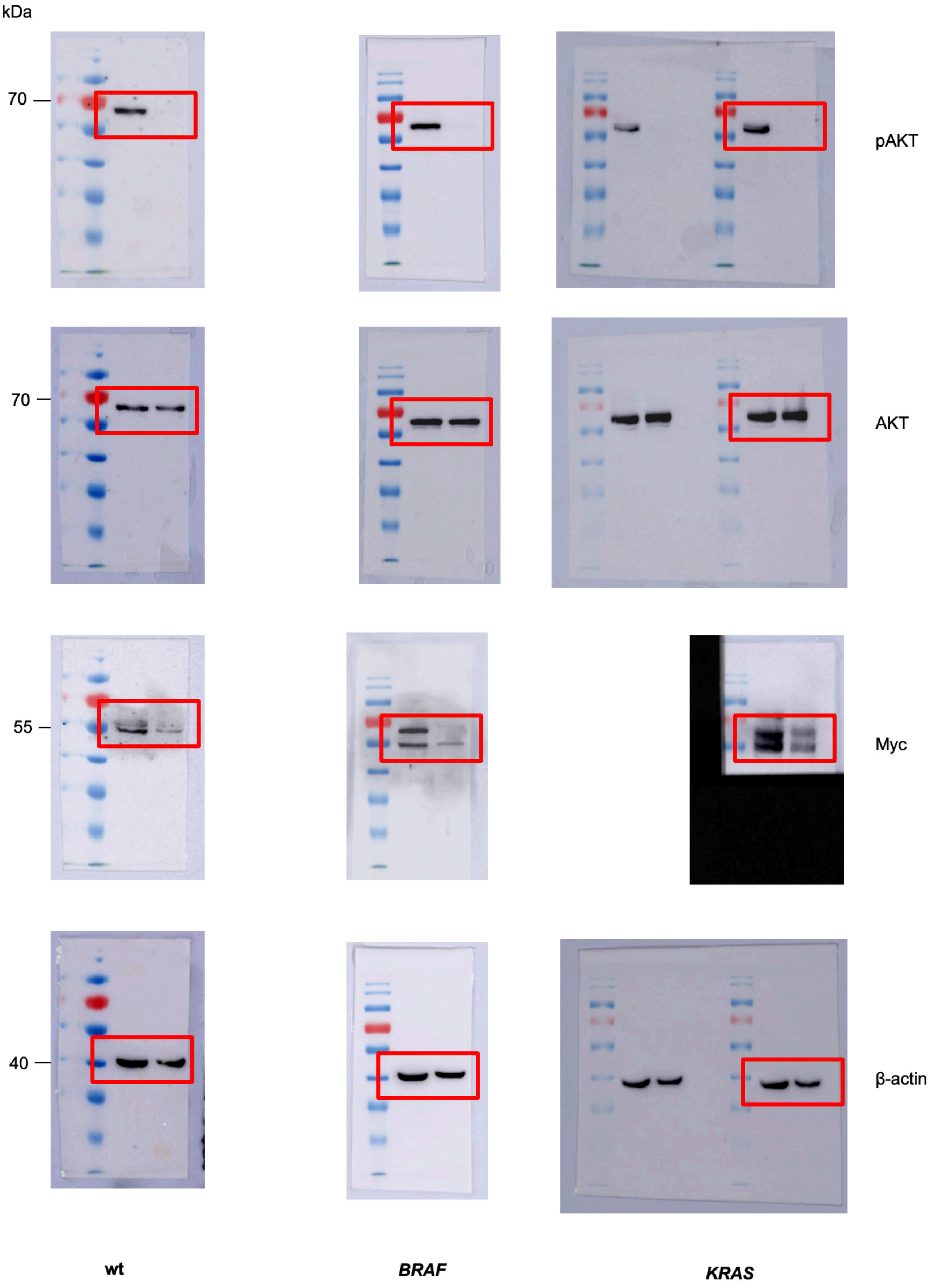

Full unedited gel for Figure S1 B

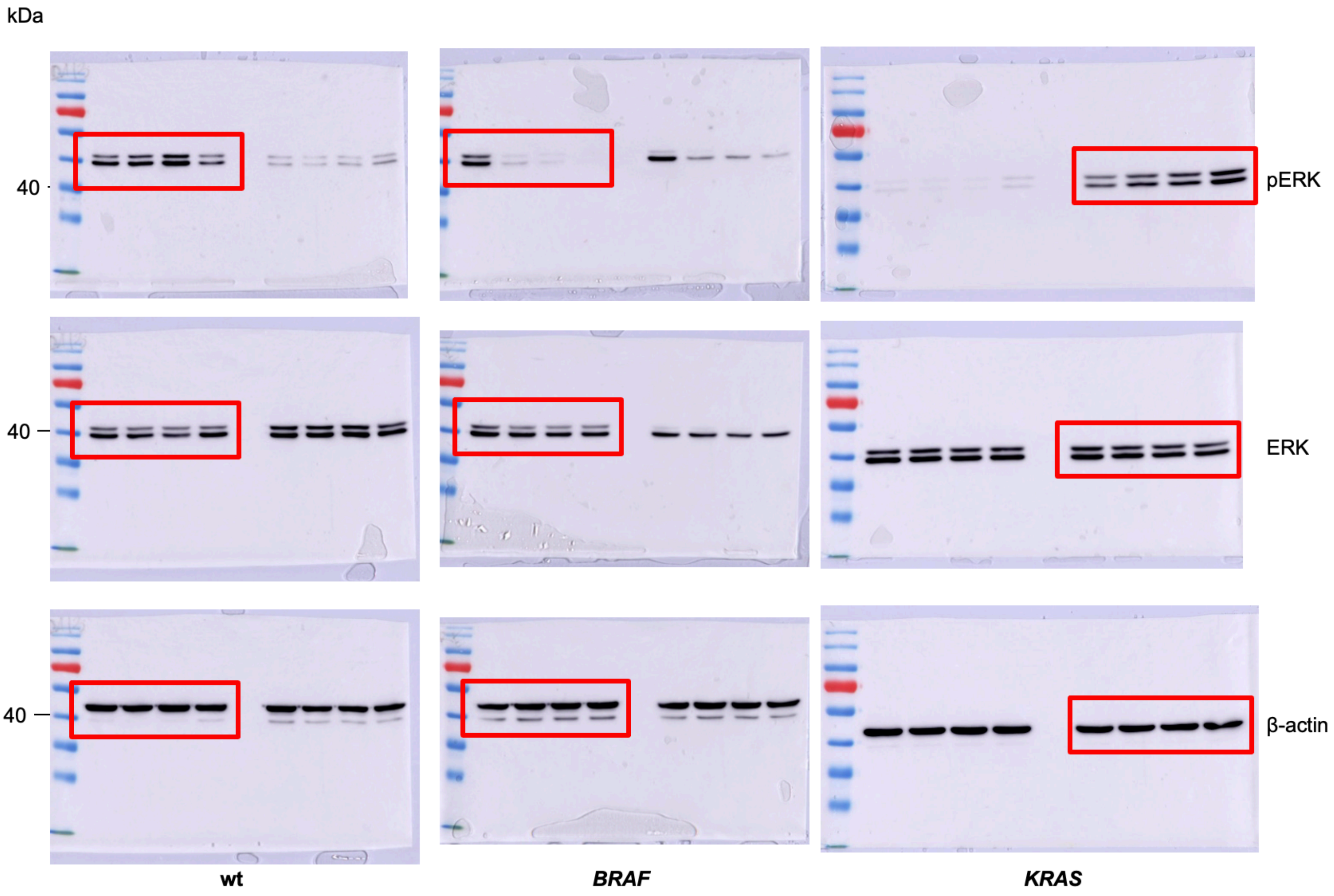

Supplement: Supplementary file 1 [file cancers-14-00673-s001.zip › cancers-1474231-supplementary/Gaggianesi et al Supplementary information cancers-1474231#2.pdf]
